# Sleep Quality Among Pilgrims at High Altitude: A Cross-Sectional Study From Gosaikunda Lake, Nepal (4380 m)

**DOI:** 10.7759/cureus.72604

**Published:** 2024-10-29

**Authors:** Barun Mahat, Bikalpa Thapa, Indrajit Banerjee, Shavana SJB Rana, Yeshashree Rajaure, Lava Shrestha, Naresh Manandhar, Bipin Shrestha, Sunil Dhungel, Tara Man Amatya, Arun Neopane

**Affiliations:** 1 Department of Clinical Physiology, Nepalese Army Institute of Health Sciences, Kathmandu, NPL; 2 Department of Pharmacology, Sir Seewoosagur Ramgoolam Medical College, Belle Rive, MUS; 3 Department of High Altitude and Mountain Medicine, 153 General Hospital, Leh, IND; 4 Department of Clinical Physiology, Maharjgunj Medical Campus, Kathmandu, NPL; 5 Department of Community Medicine, Nepalese Army Institute of Health Sciences, Kathmandu, NPL; 6 Department of Neuroscience and Physiology, Medical University of Americas, Charlestown, KNA; 7 Department of Pediatrics, Shree Birendra Hospital, Kathmandu, NPL

**Keywords:** altitude sickness, brain hypoxia, hypoxia in sleep, insomnia disorder, “nepal”, poor sleep quality, sleep initiation and maintenance disorders

## Abstract

Introduction

Nepal is a touristic country; globally, many people visit Nepal for mountaineering, trekking, sightseeing, and pilgrimages. Gosaikunda, located at an elevation of 4380 m (14,370 ft) in the Rasuwa district, Nepal, is a popular pilgrimage site. At high altitudes, hypobaric hypoxia is the primary cause of sleep disturbances and is characterized by difficulty falling asleep, frequent nighttime awakening, difficulty returning to sleep, and waking up earlier in the morning than desired, ultimately resulting in a reduction in total sleep duration and quality.

Objective

The primary objective of this study was to evaluate the quality of sleep patterns of pilgrims while undergoing acute high-altitude exposure on their journey to Gosaikunda, Nepal, which is a pilgrimage site situated at an altitude of 4380 meters (14,370 feet) above sea level.

Methodology

A cross-sectional descriptive study from August 7 to 14, 2022, was conducted among Gosaikunda pilgrims who visited the sacred lake in Rasuwa district in Nepal at an altitude of 4380 m, where weather is unpredictable and adverse climatic events are prevalent. The subjective sleep quality was evaluated by using the Athens Insomnia Scale (AIS). Individual participants rated each item (sleep symptoms) as 0 to 3, 0 = no problem, 1 = slight problem, 2 = marked problem, and 3 = very marked or no sleep at all. The total range of the score is 0 to 24, with a cutoff point score ≥ 6 being considered poor sleep.

Results

Out of 229 participants, 42 (18%), 24 (11%), and three (1%) of them experienced mild, moderate, and severe insomnia, and 160 (70%) had no sleep disturbances. Based on the Athens Insomnia Scale cutoff points, 69 (30.13%) had a score of ≥ 6, indicative of insomnia, and 160 (69.86%) had a score of less than 6, suggestive of no insomnia. Daytime sleepiness was the most common subjective sleep issue among the pilgrims suffering from insomnia (40, 57.97%) and no insomnia (96, 60%). The majority of pilgrims, 207 (99.12%), stayed overnight while ascending at Gosiakunda (4380 meters). The mean body mass index (BMI) in kg/m^2^ of pilgrims suffering from insomnia and those not suffering from insomnia was 25.29±5.3 and 24.58±4.47, respectively, with a P-value greater than 0.05. The mean age among pilgrims suffering from insomnia and pilgrims who didn’t was 41.64±13.39 and 41.64±13.44, respectively, with a P-value greater than 0.05. The majority of the pilgrims took an average of two days to reach Gosaikunda Lake, of which 207 (99.12%) remained at altitude for one night and 22 (0.96%) stayed for more than one night.

Conclusion

Acute exposure to high altitude results in frequent arousal due to hypobaric hypoxia, which in turn causes pilgrims to feel mentally and physically fatigued and somnolent due to the poor sleep they experience. An elevated BMI, advanced age, and male sex were associated with poor sleep quality after acute altitude exposure. Further research is needed to better understand the mechanisms underlying these associations and to develop effective interventions to improve sleep quality during rapid ascent.

## Introduction

Every year, thousands of people from around the world visit Nepal for trekking, climbing, sightseeing, and pilgrimages. Out of the many pilgrimage sites in Nepal, Gosaikunda Sacred Lake is one of the most popular pilgrimage sites, located at an elevation of 4380 m (14,370 ft) in the Rasuwa district situated in the Central Development Region of Nepal [1]. Every year, thousands of pilgrims visit this lake during Jania Purnima, a holy day of Hindus for spiritual and ritual fulfillment [2]. Most of the pilgrims ascend rapidly, taking around two days on average to reach the lake [1-3]. It is a noteworthy altitude gain from 1950 m to 4380 m in merely two days [4-7].

Acute altitude exposure leads to acute mountain sickness (AMS), significant sleep quality disturbances due to difficulty in the onset of sleep, and frequent awakening, which affects daytime performance and induces drowsiness the next day [8-14]. At high altitudes, the oxygen concentration in the atmosphere is low, known as hypobaric hypoxia. The predominant cause of sleep disturbances at altitudes is due to hypobaric hypoxia [15-18]. Several studies have reported that the prevalence and severity of sleep disturbances can be provoked by the rate and duration of ascent and time spent at high altitudes [7,19].

Studies conducted by Basnyat B, et al., Thapa SS, et al., MacInnis MJ, et al., and several other studies have revealed that AMS is common among the pilgrims going to Gosaikunda [1-3,20-22]. There is a dearth of data regarding the effects of acute high-altitude exposure on sleep patterns amongst the devotee population in Gosaikunda Lake, Rasuwa district in particular, and Nepal in general.

Therefore, this study aims to evaluate high-altitude sleep patterns among pilgrims at the Gosaikunda Lake in the Rasuwa district, situated in the Central Development Region of Nepal. The primary objective of this study was to evaluate the quality of sleep after acute exposure to high altitude among pilgrims traveling to Gosaikunda Lake, Rasuwa District, Nepal.

## Materials and methods

Study design and sampling method

This was a cross-sectional descriptive study conducted among the pilgrims who visited Gosaikunda Lake. The participants were selected using non-probability sampling.

Study population and sites of study

This study was conducted among pilgrims who traveled to Gosaikunda and who had voluntarily visited the health camp for medical support during the Janai-Purnima festival. Janai-Purnima is a famous Hindu festival celebrated by the Nepalese population. Janai signifies the sacred thread, and Purnima signifies the full moon. This festival is celebrated in the month of August. Thousands of tourists and local Nepalese visit the sacred Gosaikunda Lake to embrace the occasion during this festival. 

Data collection

The data was collected from August 7 to 14, 2022. This study was conducted at Gosaikunda in the Rasuwa district of Nepal, at an altitude of 4380 m (14,370 ft), where weather is unpredictable and adverse climatic events are prevalent. 

Inclusion criteria

Pilgrims who ascended to Gosiakund Lake and voluntarily visited the medical camp at Gosaikunda Lake were included in the study. The questionnaire was administered when the pilgrims reached Gosaikunda Lake, which is located at an altitude of 4380 m (14,370 ft) (Appendices 1, 2). The medical camp was located at the same altitude as the Gosaikunda Lake. Informed consent was obtained from the pilgrims before their inclusion in this study. 

Exclusion criteria

The study excluded those taking medications that could prevent high altitude sickness, those with medical comorbidities like heart disease, diabetes mellitus, restrictive and obstructive lung diseases, and those who smoked or drank alcohol. The pilgrims who hadn’t given their consent were also excluded.

Data collection tools and techniques

The data was collected after obtaining informed consent and thoroughly explaining the purpose of the study. Ethical approval was obtained from the Institutional Review Committee of the Nepalese Army Institutes of Health Sciences (Approval/Reference number: 662). The research was conducted in accordance with the Helsinki Ethical Principles for Medical Research involving Human Subjects guidelines.

Measurement of sleep quality

The quality of sleep was assessed by using the Athens Insomnia Scale (AIS). AIS is a four-likert type self-rated questionnaire used to assess subjective feelings of sleep difficulty. It contains eight different questions [22]. Individual participants rated each of the AIS items (symptoms) as 0 (no problem) to 3, indicated as 1 (slightly), 2 (markedly), and 3 (problematic or no sleep at all). The total range of the score calculated is 0 to 24. Based on AIS cutoff points of ≥6 and the International Classification of Diseases (ICD-10) criteria, individual participants were divided into two groups: those who experienced sleep disturbance (poor sleep) and those who did not suffer from sleep disturbance (normal or good sleep) [23,24]. The grading of sleep quality was categorized based on the score as an absence of insomnia if the score was 0-5, mild insomnia if 6-9, moderate insomnia if 10-15, and severe insomnia if 14-24 [25].

Data analysis procedure

Microsoft Excel 2016 (Microsoft Corporation, Redmond, Washington, United States) was used for data entry, and IBM SPSS Statistics for Windows, Version 26 (Released 2019; IBM Corp., Armonk, New York, United States) was used for the analysis of collected data. The demography and prevalence of the sleep disturbance results are presented in the form of mean-standard deviation and percentage. A chi-square test was used to find the association between demographics and insomnia and no insomnia. A value for probability less than 0.05 (p< 0.05) at the 95% confidence interval was considered statistically significant.

## Results

Out of 229 participants, 148 (64.60%) were male and 81 (35.40%) were female. The overall demographics of pilgrims are shown in Table 1. Table 2 depicts the prevalence and severity of insomnia symptoms among participants.

**Table 1:** Overall demographics of participants BMI: body mass index

Demographic profile	Number (%)
Male	148 (64.60%)
Female	81 (35.40%)
BMI in kg/m^2^ (Mean ± SD)	24.79 ± 4.8
BMI	Maximum: 48.78
	Minimum: 15.43
Age- years (Mean ± SD)	41.69 ± 13.40 years
	Maximum: 77 years
	Minimum: 16 years

**Table 2:** Prevalence of severity of insomnia symptoms among participants

Severity of insomnia	Number (%)
Normal	160 (69.90%)
Mild insomnia	42 (18.30%)
Moderate insomnia	24 (10.50%)
Severe insomnia	3 (1.30%)

Based on the AIS cutoff points, 69 (30.13%) had a score of ≥ 6, indicative of pilgrims suffering from insomnia (complaints of sleep disturbance), and 160 (69.86%) had a score of less than 6, suggestive of no insomnia (no sleep complaints). Among pilgrims suffering from insomnia, 35 (50.7%) were male and 34 (49.3%) were female. The mean AIS score of pilgrims with and without insomnia was 8.94±2.76 and 2.31±.92, respectively. The BMI of pilgrims with insomnia was 25.29±5.3 kg/m^2^ more than that of pilgrims without insomnia, 24.58±4.47 kg/m^2^. This finding is, however, not statistically significant. The mean age of pilgrims suffering from insomnia was 41.64±13.39 years, slightly higher than pilgrims who didn’t suffer from insomnia, 38.59±12.95 years. At high altitudes, the prevalence of subjective symptoms of insomnia was found more in pilgrims with a higher BMI and age (Table 3).

**Table 3:** Demographics of pilgrims suffering from insomnia and those who didn’t suffer from insomnia AIS: Athens Insomnia Scale; BMI: body mass index * P-value- significant

Demographics	Pilgrims with insomnia	Pilgrims without insomnia	P-value significances <0.05
AIS score (Mean ± SD)	8.94±2.76	2.31±1.92	
	Maximum score: 19	Maximum score: 5	
	Minimum score: 6	Minimum score: 0	
BMI in kg/m^2^ (Mean ± SD)	25.29±5.3	24.58±4.47	0.609
	Maximum (BMI): 46.72	Maximum (BMI): 48.78	
	Minimum (BMI): 17.71	Minimum (BMI): 15.43	
Age- years (Mean ± SD)	41.64±13.39	38.59±12.95	0.490*
	Maximum (Age): 76	Maximum (Age): 77	
	Minimum (Age): 16	Minimum (Age): 19	

Based on the number of participants who rated their sleep as a "slightly, markedly, or problematic (sleep complaint)," we calculated the cumulative sleep complaints among pilgrims suffering from insomnia and participants without insomnia. The complaints of subjective sleep issues among the pilgrims suffering from insomnia were daytime sleepiness followed by difficulty in sleep onset and frequent awakening at night, as shown in Table 4. Similarly, Table 4 illustrates the complaints of subjective sleep issues among pilgrims with insomnia and those without, which included daytime sleepiness, frequent nighttime awakenings, and difficulty in initiating sleep.

**Table 4:** Prevalence of sleep complaints among pilgrims suffering from insomnia and those who didn’t suffer from insomnia AIS: Athens Insomnia Scale

AIS Items	Pilgrims with insomnia (N = 69)		Pilgrims without insomnia (N = 160)	
	With symptoms (slightly, markedly, or problematic)	Normal (without symptoms)	With symptoms (slightly, markedly, or problematic)	Normal (without symptoms)
Sleep induction	36(52.17%)	33(47.82%)	79(49.37%)	81(50.62%)
Awakening during night	34(49.27%)	35(50.72%)	90(56.25%)	70(43.75%)
Early rising	21(30.43%)	48(69.56%)	77(48.12%)	83(51.87%)
Total sleep duration	25(36.23%)	44(63.76%)	78(48.75%)	82(51.25%)
Overall sleep quality	25(36.23%)	44(63.76%)	62(38.75%)	98(61.25%)
Wellbeing during day	24(34.78%)	45(65.52%)	64(40%)	96(60%)
Functional capacities during the day	31(44.92%)	38(55.07%)	74(46.25%)	86(53.75%)
Sleepiness during the day	40(57.97%)	29(42.20%)	96(60%)	64(40%)

The majority of pilgrims take an average of two days to reach Gosaikunda Sacred Lake. While ascending to the top, out of 229 participants, 207 (99.12%) of pilgrims slept one night at the high altitude, and 22 (0.96%) spent more than one night. However, out of 69 pilgrims suffering from insomnia, 57 (82.26%) spent only one night at altitude, and 12 (17.39%) spent more than one night. Out of 160 pilgrims who were not insomniacs, 150 (93.75%) spent the night, and 10 (6.25%) spent more than one night.

## Discussion

In addition to other symptoms of high-altitude sickness, such as headaches, fatigue, nausea, and digestive problems, sleep disturbance is also a common symptom that occurs at high altitudes. Sleep disorders, particularly insomnia, have been characterized by difficulty in sleep onset, frequent awakenings at night, early rising, a reduced sleep duration associated with poor daytime performances, and a sense of unwellness [26,27]. 

In this study, the subjective sleep problems among the pilgrims suffering from insomnia were daytime sleepiness, followed by difficulty in sleep onsets, reduced daytime performance, reduced total sleep duration, and overall sleep quality. In the studies by Szymack et al. and Brian SZ et al., subjective insomnia symptoms were noted in 47% and 63.3% of the total sample after acute high-altitude exposure due to frequent arousals during the night, difficulty in sleep induction, temperature-related discomfort, and breathing difficulties [8,28]. In this study, we observed that 30.13% of pilgrims had subjective insomnia symptoms after acute altitude exposure.

In a study conducted by Tang XG et al. on sleep quality changes in insomniacs and non-insomniacs after acute altitude exposure, 20.98% of the total sample experienced poor sleep quality at 500 m, but after acute exposure to an altitude of 3700 m, the occurrence of subjective insomnia symptoms significantly increased in both insomniac and non-insomniac groups. In both groups, poor sleep quality was due to repeated arousal at night, followed by difficulty in sleep induction and daytime sleepiness [29]. In our study, the subjective sleep problems among non-insomniac pilgrims were daytime sleepiness followed by frequent awakening at night and difficulty in the onset of sleep. 

Several sleep studies conducted on mountaineers, trekkers, and native populations have reported that sleep problems were due to difficulties in sleep induction, increased awakenings, and frequent brief arousals at night due to marked nocturnal hypoxemia and periodic breathing after acute altitude exposure [30,31].

In this study, the majority of pilgrimages took an average of two days to reach Gosaikunda Sacred Lake. The conditions align with studies conducted by Zafran et al. and Basnyet et al. [20,1]. In their study, acute mountain sickness (AMS) was reported in 68% and 29% of pilgrims, who took an average of around two days to reach Gosaikunda Lake. According to Luke AM et al., to minimize the risk of AMS, individuals should not increase their sleeping altitude by greater than 300-500m per day while over a 3000 m elevation and include a rest day every three or four days when trekking above 3000 m [32]. Several studies have reported an increase in sleep problems with increasing severity of altitude exposure as well [33,34].

In accordance with studies conducted by Bian SZ et al. [28] and Hargens TA et al. [35], we also observed that an increased BMI and age were associated with the occurrence of sleep disturbances at high altitudes in our study. Further research is needed to better understand the mechanisms underlying these associations and to develop effective interventions to improve sleep quality during rapid ascent.

The strength of the study was that the data was collected from the pilgrims who visited Gosaikunda Lake in the Rasuwa district located in the Central Development Region of Nepal, which is situated at an altitude of 4380 m where weather is unpredictable and adverse climatic events are prevalent and is challenging to conduct research at such an altitude. Our study was limited by our inability to evaluate the quality of sleep prior to the high-altitude pilgrimage.

## Conclusions

Acute exposure to high altitude leads to frequent arousal due to hypobaric hypoxia, which in turn causes pilgrims to feel mentally and physically fatigued and somnolent due to the poor sleep they experience. An elevated BMI, advanced age, and male sex were associated with poor sleep quality after sudden high altitude exposure. Further studies are required to better understand the mechanisms and pathophysiology underlying these associations and to develop effective interventions to improve sleep quality at high altitudes. 

## Appendices

Appendix 1 (original questionnaire)

साधारण जानकारी

१. मिति

२. नाम थर

३. उमेर/लिङ्ग

४. स्थाई ठेगाना

५. हाल बसोवास गर्ने ठेगाना

६. उँचाई

७. वजन

८. सर्वेक्षण गरेको स्थान र Altitude

९. Medical History

​क. ब्लड प्रेसरः

​ख. मदिरा सेवनः छ / छैन

​ग. चुरोट सेवनः छ / छैन

​घ. औषधि सेवनः प्रेसर / चिनी / Cholesterol / High Altitude / अन्य भएः _____________

​ङ. पहिले High Altitude मा गएको छ / छैन

​च. High Altitude Illness पहिले भएको छ / छैन

​छ. पहिले निद्रा सम्बन्धी केही समस्या थियो / थिएन

१०. Altitude Exposure History (Acclimatization)

क्र.सं

वासस्थान (उँचाई)

समय अवधि

१

२

३

४

५

११. Athens Insomnia Scale (AIS) 

१. निद्रा पर्न केहि समस्या

        ०                                  १                              २                              ३

केही समस्या नभएको            केही ढिला                   धेरै ढिलो                 निद्रै लाग्दैन

२. राती निद्रा खुल्ने समस्या

        ०                                  १                              २                              ३

केही समस्या नभएको           न्युन समस्या              उल्लेख्य समस्या           निद्रै लाग्दैन

३. बिहान ब्युझिने समय

        ०                                  १                              २                              ३

पहिले जस्तै           साधारण भन्दा केहि अगाडी    साधारण भन्दा धेरै अगाडी   निद्रै लाग्दैन

४. सुतेको अवधि

        ०                                  १                              २                              ३

पुग्दो छ                      अली-अली नपुग छ          असाध्यै नपुग छ              निद्रै लाग्दैन

५. निद्राको गुणस्तर

        ०                                  १                              २                              ३

सन्तोषजनक                 अली-अली कम छ            धेरै कम छ                   निद्रै लाग्दैन

६. दिनको समयमा स्वास्थ्यस्थिती

        ०                                  १                              २                              ३

ठीक छ                        अली-अली खराब छ         धेरै खराब छ              अती नै खराब छ

७. दिनमा काम-काजको अवस्था

        ०                                  १                              २                              ३

ठीक छ                        अली-अली खराब छ         धेरै खराब छ              अती नै खराब छ

८. दिनमा निद्रा लाग्छ

        ०                                  १                              २                              ३

लाग्दैन                              थोरै लाग्छ                  धेरै लाग्छ               अती नै लाग्छ

Appendix 2 (English translation)

1. Date:

2. Name:

3. Age:

4. Permanent Address:

5. Temporary Address:

6. Height(cm):

7. Weight(kg):

8. Data Collection Altitude:

9. Medical History

9.1 H/O Blood pressure: Yes/ No

9.2 H/O Alcohol: Yes/No

9.3 H/O Smoking: Yes/No

9.4 H/O Respiratory Diseases: Yes/No

9.5 H/O Cardiovascular Diseases: Yes/No

9.6 H/O Pills/ Medication: Hypertension/ Diabetic Mellitus/Cholesterol/High Altitude/Others

9.7 Past Travel H/O Altitude: Yes/ No

9.8 Past H/O of High Altitude Illness: Yes/ No

9.9 Past H/O Sleep Disorder: Yes/ No

10 . Altitude Exposure History (Acclimatization)

Serial no.                 Sleep-altitude             Duration of sleep-altitude

11. Athens Insomnia Scale (AIS) 

Please, circle or tick the appropriate number) the items below to indicate your estimate of any sleep difficulty you might have experienced.

AIS items 

1. Sleep induction (the time it takes you to fall asleep after turning off the lights)

0                                           1                                           2                                                   3

No problem         Slightly delayed                     Markedly delayed            Very delayed or did not sleep at all

2. Awakening during the night 

0                                           1                                           2                                                   3

No problem                Minor problem                  Considerable problem   Serious problem or did not at all

3. Final awakening earlier than desired 

0                                           1                                           2                                                   3

No earlier                   A little earlier                   Markedly earlier          Much earlier or did not sleep at all

4. Total sleep duration

0                                           1                                           2                                                   3

Sufficient            Slightly insufficient             Markedly insufficient            Very insufficient or did not sleep at all

5. Overall quality of sleep

0                                           1                                           2                                                   3

Satisfactory        Slightly unsatisfactory      Markedly unsatisfactory      Very unsatisfactory or did not sleep at all

6. Sense of well-being during the day

0                                           1                                           2                                                   3

Normal                Slightly decreased            Markedly decreased                       Very decreased

7. Functioning (physical and mental) during the day 

0                                           1                                           2                                                   3

Normal                Slightly decreased            Markedly decreased                    Very decreased 

8. Sleepiness during the day

0                                           1                                           2                                                   3

None                                 Mild                                  Moderate                                    Intense 
